# Institutional Notes and News

**Published:** 1911-05-27

**Authors:** 


### Institutional Notes and News.

On Tuesday the Duke of Connaught visited the Cancer Hospital, Fulham Road, S.W., for the purpose of opening the new Cancer Research Institute recently built there. The ceremony was to have taken place a month ago, but was postponed in consequence of the indisposition of his Royal Highness. At three o'clock the Duke and Duchess arrived at the hospital, and were received at the entrance and conducted to the Board Room by the Earl of Northbrook (President) and Colonel Marsdin Newton (Chairman of the House Committee). After the presentation of members of the committee and staff of the hospital, the President made a short statement, after which his Royal Highness delivered an address. Her Royal Highness the Duchess was then presented with a bouquet, after which the royal party visited some of the wards, operating theatre, etc. After this their Royal Highnesses passed into the grounds and paid a brief visit to the nurses' home. The new Research Institute was next reached, and the architect (Mr. Graham) presented the Duke with a silver key. Unlocking the door the Duke declared the Institute opened, and their Royal Highnesses then inspected it in the company of the director and other members of the staff. The band of the Coldstream Guards under Dr. J. Mackenzie Rogan played a selection of music in the grounds of the hospital, which were visited, as were also the wards, by a large number of interested spectators after the departure of the Duke and Duchess.

**Duke of Connaught Visits the Cancer Hospital.**

The new Research Institute thus duly opened has been planned in accordance with the requirements of the scheme of research adopted by the hospital, and provides for study in every branch of medical science bearing on the subject of cancer. The main entrance on the ground floor leads to a spacious hall, around which are disposed the various offices of administration. The Director's room, adjoining which is his laboratory; the office and attendant's room; and a large reading and reference room are on the first floor. The provision of cancer literature will be extremely full and complete. In the basement there is a machine room, installed with machinery and electric plant, adjoining it a large cold store and refrigerator, and leading off the machine room is a centrifuge room. On this floor are also placed the laboratory for experimental electro-radio physics, and in connection with it a large dark room and photographic department. A special laboratory has been set aside for preparatory chemistry, equipped with mills, presses, combustion furnace, and an apparatus for the preparation of distilled water. There is also a room for sterilisation of culture media, an underground laboratory for working at an equable temperature, and workshops, stores, and other offices. The upper part of the building is mainly devoted to the research laboratories, ten in number; five of these are set apart for the important branches of chemistry and physics. The same arrangement has been maintained as in the lower part; the laboratories, which for the most part intercommunicate, also open into the central hall. The Institute is lighted throughout with electricity, and it is also thoroughly equipped with gas and hot and cold water, and is heated by radiators. It is being furnished with the most recent scientific apparatus, and is adequate for the most advanced research.

**The Research Institute Described.**

It will not be forgotten, as the Chairman (Dr. A. P. Beddard) pointed out, that the annual dinner of the West London Hospital and Post-Graduate College, which took place last week, is the prelude to the big Coronation dinner at the White City, over which, as The Hospital has announced already, the Duke of Abercorn will preside in a month's time. The needs and position of the West London Hospital, to which Dr. Beddard referred at the dinner, were dealt with exhaustively in this journal on April 29. The rest of his speech was concerned largely with the probable effects of the Government's insurance proposals upon the medical profession. Some thought, he said, it meant the end of voluntary hospitals; at any rate it would remove a great burden of anxiety from the minds of certain gentlemen who tried to make the income of the West London Hospital adequate to its needs. He then went on to say that if the medical profession combined together they could make a very good bargain. The dinner was made exceptionally lively by the opportunities these inspiring topics afforded to the speakers.

**West London Hospital Dinner.**

The expediency of effecting improvements at the Bingley Cottage Hospital (Yorks) has recently engaged considerable attention in the West Riding town, and it is pleasing to note that as a result of careful deliberations, the local committee has determined to proceed with the work of extending the hospital. The institution was founded as a memorial of Queen Victoria's Jubilee in 1887, and the suggestion that the extension of the hospital should represent a permanent memorial to the late King Edward VII. was readily approved at a recent town's meeting, so that when the work is completed the building will stand as a joint memorial to our late ruler and his revered mother. The accommodation has for some time past been taxed to some extent, and plans which have been obtained for the proposed extension cover the provision of about double the present number of beds in both the male and female wards, and also allow for increased staff accommodation to be given. The work of extension will not take place all at one time—the financial requirements doubtless not being immediately forthcoming—but the committee decided on Thursday last to go forward with half the scheme, which means that the present men's ward will be doubled in size and additional bedrooms and staff accommodation provided above. The enlarged ward will then become the female ward. The effect of the extension will be to give accommodation for seven more beds in the ward, and in addition two smaller rooms, which have been hitherto utilised as sleeping apartments for members of the staff, will be available for the use of patients. The cost of the present work is estimated at £500. Altogether the committees of the West Riding hospitals are displaying the utmost zeal in the conduct of the work to which they have turned their hands, and it is safe to assert that the better facilities and increased accommodation which are in prospect at the institutions in various parts of the Riding will prove, when carried into effect, to be of inestimable value not only to the sick, but to the nursing staff as well.

**Yorkshire Cottage Hospital Extended.**

---

Every seat was sold at last week's matinée at the Playhouse in aid of the Miller General Hospital, which is expected to receive nearly £500.

